# TACI Expression and Signaling in Chronic Lymphocytic Leukemia

**DOI:** 10.1155/2015/478753

**Published:** 2015-04-09

**Authors:** Antigoni Mamara, Anastasios E. Germenis, Maria Kompoti, Maria Palassopoulou, Eudokia Mandala, Anastasia Banti, Nikolaos Giannakoulas, Matthaios Speletas

**Affiliations:** ^1^Department of Immunology & Histocompatibility, Faculty of Medicine, School of Health Sciences, University of Thessaly, Biopolis 3, 41500 Larissa, Greece; ^2^Department of Hematology, Faculty of Medicine, School of Health Sciences, University of Thessaly, Biopolis 3, 41500 Larissa, Greece; ^3^Fourth Department of Internal Medicine, Ippokration Hospital, Aristotle University of Thessaloniki, Konstantinoupoleos 49, 54642 Thessaloniki, Greece; ^4^Department of Hematology, Papageorgiou General Hospital, Periferiaki Odos Thessalonikis, Nea Efkarpia, 56429 Thessaloniki, Greece

## Abstract

TACI is a membrane receptor of BAFF and APRIL, contributing to the differentiation and survival of normal B cells. Although malignant B cells are also subjected on TACI signaling, there is a remarkable intradisease and interindividual variability of TACI expression in B-cell malignancies. The aim of our study was to explore the possible role of TACI signaling in the biology of chronic lymphocytic leukemia (CLL), including its phenotypic and clinical characteristics and prognosis. Ninety-four patients and 19 healthy controls were studied. CLL patients exhibited variable TACI expression, with the majority of cases displaying low to undetectable TACI, along with low to undetectable BAFF and increased APRIL serum levels compared to healthy controls. CLL cells with high TACI expression displayed a better survival capacity in vitro, when cultured with BAFF and/or APRIL. Moreover, TACI expression was positively correlated with the presence of monoclonal gammopathy and inversely with CD11c expression. Therefore, our study provides further evidence for the contribution of BAFF/APRIL signaling to CLL biology, suggesting also that TACI detection might be useful in the selection of patients for novel targeting therapeutic approaches.

## 1. Introduction

B-cell chronic lymphocytic leukemia (CLL) is the most common hematologic malignancy in the Western world. The disease is characterized by proliferation and accumulation of CD5^+^, CD19^+^, CD20^low^, and surface immunoglobulin (Ig)^low^ expressing B cells, with balanced homeostasis in patients with stable lymphocyte count, and imbalanced homeostasis in patients with increasing lymphocyte count [1, 2]. CLL is an incurable and heterogeneous disease, clinically divided into two major entities based on the presence or absence of somatic mutations in the variable region of the immunoglobulin heavy-chain (IGHV) gene [3, 4]. There is no unique genetic defect identified in CLL, although over 80% of the patients present with cytogenetic lesions [5, 6].

Signals delivered by direct cell contact or soluble factors, which may or may not occur concurrently with B-cell-receptor engagement, propagate the growth of CLL cells. The disruption of this cross talk is an attractive novel strategy for treating the affected patients [7, 8]. Recent studies have demonstrated that tumor necrosis factor (TNF) superfamily members may contribute to the enhanced survival potential of malignant lymphocytes. In particular, neoplastic B cells express variable protein levels of B-cell-activating factor (BAFF) receptor (BAFFR, encoded by* TNFRSF13C*), transmembrane activator, and calcium modulator and cyclophilin ligand interactor (TACI, encoded by* TNFRSF13B*), and B-cell maturation antigen (BCMA, encoded by* TNFRSF17*). When these receptors are bound to BAFF (also known as BLyS, TALL-1, zTNF4, and THANK) and/or A proliferation-inducing ligand (APRIL), which are produced by antigen presenting cells (APCs), stromal endothelial cells, nurse-like, and/or malignant cells, they can evade apoptosis and promote tumor cell survival in vitro [9–14]. BAFF and APRIL bind TACI and BCMA, while BAFF, but not APRIL, binds BAFFR [15, 16]. Normally, BAFF and APRIL are required for the development of most but not all normal mature B-cell populations found in the periphery [17, 18].

BAFF and APRIL in CLL cells induce activation of the nuclear factor-kappaB1 (NF-*κ*B1), namely, the classical NF-*κ*B pathway, through BCMA and TACI. However, BAFF, but not APRIL, also induces the activation of NF-*κ*B2/p100, namely, the alternative NF-*κ*B pathway, apparently through binding to BAFFR [12, 13]. At the same time, the blocking of the classical NF-*κ*B pathway blocked the capacity of BAFF to support neoplastic B-cell survival, without any effect on the survival of normal B cells, in which the activation of alternative NF-*κ*B pathway prevails [13]. Interestingly, CLL cells may variably express TACI and very low to undetectable BCMA [9, 11, 13, 19], but all cells express BAFFR with a rather lower intensity compared to normal B cells [9, 11, 13, 14, 20, 21]. These findings suggest that BAFF/APRIL signaling in neoplastic CLL cells might be important.

Considering that CLL is characterized by a variable clinical and laboratory presentation and prognosis and that the majority of previous studies have enrolled a few CLL patients (ranged from 10–25), the contribution of TACI expression in the disease phenotype remains as yet obscure. The clarification of its role might be of outstanding interest, since novel anti-BAFF/APRIL treatments have recently been approved for the management of patients with B-cell-mediated autoimmune diseases [22, 23], suggesting that the targeting of TACI might be also a therapeutic candidate in B-cell malignancies [24–26]. Therefore, the aim of this study was to investigate the contribution of TACI signaling to the biological, clinical, laboratory, and prognostic characteristics of CLL.

## 2. Material and Methods

### 2.1. Subjects

Ninety-four CLL patients (male/female: 53/41, mean age: 68.6, range: 39–87), diagnosed by standard criteria [2], were enrolled in the study. Among them, 54 were analyzed at diagnosis, 21 had not received any treatment in the past, and the rest 19 patients were analyzed at relapse. For the majority (90 out of 94) of patients, flow cytometric and molecular analyses were performed in peripheral blood (PB), in 4 patients in bone marrow (BM), and in 3 patients, simultaneously, in both PB and BM. Moreover, in 19 healthy individuals (male/female: 7/12, mean age: 62.8, range: 29–84) a detailed analysis of TACI expression on B-cell subpopulations and measurement of BAFF and APRIL serum levels, as described below, was also performed. No patient or healthy individual was receiving any chemotherapy and/or immunosuppressive treatment over the last six months before enrollment.

Medical records of all CLL patients were reviewed and data regarding the frequency and the location of infections, the presence of autoantibodies and autoimmune manifestations, the presence of hypogammaglobulinemia or monoclonal M component, and treatment modalities (for patients at relapse) were recorded. Data from conventional and/or molecular cytogenetic analyses and ZAP-70 expression were not available for all patients at enrollment and for this reason they were not included in the statistical analysis.

The study was conducted in accordance with the principles of the Helsinki declaration and was approved by the Institutional Review Board. All patients and healthy control agreed by written informed consent and the procedures followed were in accordance with institutional guidelines.

### 2.2. Immunophenotyping Studies

Immunophenotyping was performed by flow cytometry on Coulter FC-500 instrument (Epics XL-MCL, 4 color analysis, Beckman-Coulter/BC, Hialeah, FL, USA) using a multistaining protocol and commercially available reagents. Mouse anti-human immunoglobulin G (IgG) monoclonal antibodies were used to detect in patients molecules that reacted with CD3 (clone UCHT1), CD19 (clone J4.119), CD45 (clone J.33), CD5 (clone BL1a), CD20 (clone B9ER), CD23 (clone 9P25), CD38 (clone T16), CD43 (clone DFT1), CD10 (clone ALB1), CD11c (clone BU15), CD79b (clone CB 3-1), and FMC7 (clone FMC7). All the above antibodies were purchased by BC and were conjugated with the appropriate fluorochrome (fluorescein isothiocyanate, FITC; phycoerythrin, PE; PE-cyanine5 PE-Cy5). The clonality of B cells was assessed using rabbit anti-human polyclonal antibodies (anti-kappa light chains FITC, anti-lambda light chains PE) purchased by Dako (High Wycombe, UK). The percentage of fluorescent cells and the mean fluorescence intensity (MFI) were determined in each case corrected for background fluorescence, using FITC, PE, and PE-Cy5-labelled control IgG antibodies.

In healthy individuals, peripheral blood samples were analyzed in order to indicate the different white blood cell subpopulations. Appropriate mouse anti-human IgG monoclonal antibodies were used to detect molecules reacting with CD3 (clone UCHT1), CD4 (clone 13B8.2), CD8 (clone B9.11), CD19 (clone J4.119), and CD45 (clone J.33), all purchased by BC and conjugated with the appropriate fluorochrome (FITC, PE, PE-Cy5, and ECD). The different cell populations were gated using forward and side light scatter characteristics, as well as using the expression of specific markers. The percentage of fluorescent cells and the MFI were determined as above.

The expression of TACI on CLL cells was determined on 1 × 10^5^ cells, after erythrocyte lysis (Versalyse, BC) and washing with 1x phosphate buffered saline (PBS). Afterwards, the cells were incubated with 3% normal rat blocking serum (eBioscience, San Diego, USA) at 4°C for 20 min and, subsequently, were labeled with anti-TACI (clone 1A1, PE conjugated; Abcam, Cambridge, UK), anti-CD19 (FITC conjugated, BC), and anti-CD45 (PE-Cy-5 conjugated, BC) monoclonal antibodies, for 15 min at room temperature. The percentage of TACI-positive B cells and their MFI were corrected for background fluorescence using isotype control IgG2a-PE antibody (eBioscience). Ten CLL patients exhibiting either high or very low to undetectable TACI by flow cytometry were also analyzed for IgD and CD27 expression, using a polyclonal anti-IgD antibody (clone F0189, FITC conjugated, DAKO) and a monoclonal anti-CD27 antibody (clone 1A4CD27, PE-Cy-5 conjugated, BC). The expression of TACI on B-cell subpopulations of 19 healthy individuals was determined by using the monoclonal antibodies anti-CD19 (ECD conjugated), anti-TACI (PE conjugated), anti-IgD (FITC conjugated), and anti-CD27 (PE-Cy-5 conjugated).

Taking into account the high total number of patients and controls, whole blood and/or bone marrow samples were analyzed in batches of 2–10 samples per day in order to minimize the day-to-day variation, especially for the MFI measurement.

### 2.3. *TNFRSF13B*/TACI mRNA Expression

PB or BM samples from 19 patients with CLL exhibiting a proportion of neoplastic cells more than 80%, as well as CD19^+^ B cells from 2 healthy individuals, isolated by magnetic sorting using the Easy Sep Kit on a RobosepTM instrument (STEMCELL Technologies Inc., Vancouver, Canada), were subjected to total RNA extraction. The latter was performed using TRI (Ambion, Life Technologies, Thessaloniki, Greece), according to manufacturer's instructions. Complementary DNA (cDNA) was reversed transcribed from 1 mg of RNA, using a random 6-mer oligonucleotide primer (50 pmol/*μ*L) (F. Hoffmann-La Roche, Basel, Switzerland) and M-MLV reverse transcriptase (Invitrogen, Life Technologies, Thessaloniki, Greece), according to manufacturer's instructions. The mRNA levels of* TNFRSF13B/TACI* were determined in quantitative real-time reverse-transcriptase PCR (qRT-PCR) reaction using Platinum-SYBR-Green PCR Supermix (Invitrogen, Life Technologies), in the automated thermocycler RotorGene 6000 (Corbett Life Science, Sydney, Australia). The beta-2-microglobulin (B2M) gene was used as endogenous control for sample normalization (reference gene). An 1/20 aliquot of the cDNA reaction product was used in duplicate qRT-PCR reactions, and all measurements were averaged. The primers for the amplification of* TNFRSF13B/TACI* were designed with the aid of Oligo 6.0 software (NBI, Plymouth, MN, USA) and, along with the thermocycling PCR conditions, are presented in Table 1. The efficiency of each qRT-PCR reaction ranged between 0.9 and 1.05. In order to verify the specificity of the PCR products, melting curve analysis was performed from 65°C to 95°C with 0.1°C per sec intervals and stepwise fluorescence acquisition. Relative quantification and calculation of the range of confidence were performed using the comparative ΔΔ^CT^ method, as described [27]. The relative expression of each gene is presented as a multiple of the respective gene expression in isolated B cells of a healthy individual.

### 2.4. Mutational Status of IGHV Gene Rearrangements

Genomic DNA was extracted from PB or BM, using the QIAamp DNA Blood Mini Kit (Qiagen, Crawley, UK), according to the manufacturer's instructions. The IGHV mutational status was assessed by a 2-step seminested PCR reaction as previously described [28], with some modifications (Table 1). All reactions were performed in a PCR-engine apparatus PTC-200, MJ-Research (Watertown-Massachusetts) and the PCR products were analyzed in 3% TBE agarose gels, stained with ethidium bromide and visualized under UV light. After the identification of the a clonal IGHV rearrangement, the amplified PCR products were purified with QIAquick gel extraction kit (Qiagen) and direct sequenced using an ABI Prism 310 Genetic Analyzer (Applied Biosystems, Foster City, CA) and a Big Dye Terminator DNA sequencing kit (Applied Biosystems). The sequences were compared with the sequence of the germline variable (V) region, as presented in the National Center for Biotechnology Information website (http://www.ncbi.nlm.nih.gov/igblast/), using the Basic Local Alignment Search Tool (IgBLAST). The presence of IGHV somatic hypermutation (SHM) is defined as greater or equal to 2% different from the germline V gene sequence (mutated, M-CLL), whereas less than 2% difference is considered evidence of no SHM (unmutated, U-CLL).

### 2.5. Functional Assays and Study of Apoptosis

PB mononuclear cells from six patients with CLL, exhibiting high (>40%, 3 patients) or very low to absent (<10%, 3 patients) TACI expression, were isolated by density gradient centrifugation and diluted with Iscove's Modified Dulbecco's Medium (IMDM, Life Technologies) supplemented with 10% fetal bovine serum (FBS), at a concentration of 8 × 10^5^ cells/mL. Afterwards, aliquots of 0.5 mL of cells were plated on a 96-well plate and stimulated by either 1 *μ*g/mL of recombinant BAFF (R&D Systems, Mineapolis, USA), or 200 ng/mL APRIL (R&D Systems), or combinations of the above, diluted into 0.1 mL IMDM. Another aliquot of 0.5 mL of cells supplemented with 0.1 mL IMDM without any stimulus was used as internal control. Samples were then incubated at 37°C in the presence of 10% CO_2_ for 24 h. Subsequently, apoptosis was measured by flow cytometry using an Annexin V-FITC/7-AAD (7-AAD) kit (BC), according to the manufacturer's instructions.

### 2.6. Quantification of Soluble BAFF and APRIL

Soluble BAFF and APRIL levels were determined in the serum of 56 patients with CLL and 19 healthy individuals, using the BAFF Soluble (human, hypersensitive) ELISA Kit (Adipogen, Liestal, Switzerland) and APRIL ELISA kit (IBL, Minneapolis, USA), respectively, according to manufacturers' instructions.

### 2.7. Measurement of Immunoglobulin Levels

For the determination of the IgG, IgM, and IgA levels, we used commercially available immunonephelometric assays (Immulite-2000, Siemens Medical Solutions, Llanberis, Gwynedd, UK), with N antiserum to human IgG (product code OSAS), N antiserum to human IgA (OSAT09), N antiserum to human IgM (OSAS09), and the N protein standard SL (OQIM13), according to the manufacturer's instructions.

### 2.8. Statistical Analysis

Categorical variables were analyzed with Fisher's exact test. Normality of continuous variables was assessed with Kolmogorov-Smirnov test. Normally distributed data were analyzed with Student's *t*-test. Skewed data were analyzed with Mann-Whitney test. Survival analysis was performed with Kaplan-Meier procedure and survival rates were compared with log-rank test. Logistic regression models and Cox proportional hazards regression models were fitted as appropriate. Data analysis was performed with SPSS 17.0 (IBM Corporation, NY, 2008). Graphs were made on Excel and Prism 6 for Mac. For all analyses, alpha was set at 0.05 (2-sided).

## 3. Results

### 3.1. Clinical and Laboratory Findings of CLL Patients and Controls

**Figure 1:**
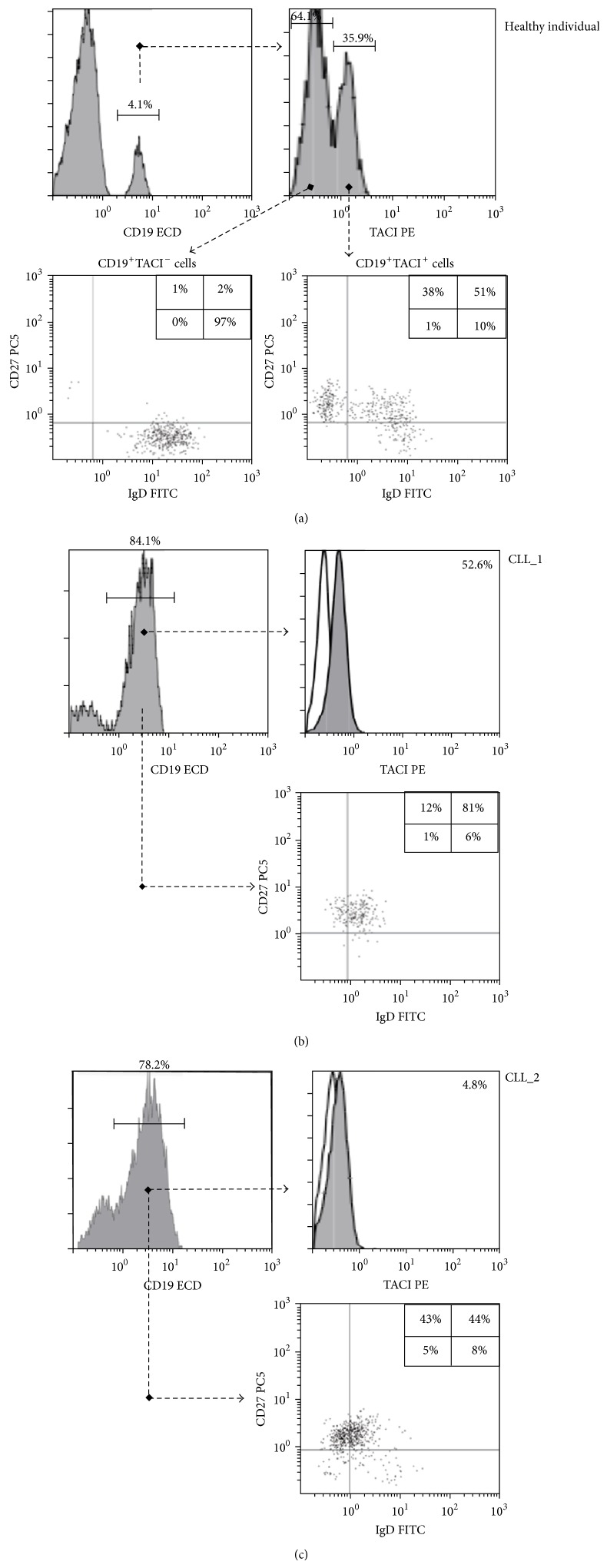
TACI along with CD27 and IgD expression on B cells established by flow cytometry in a healthy individual and 2 patients with CLL. TACI is expressed only on normal switched and nonswitched memory B cells (CD27^+^IgD^−^ and CD27^+^IgD^+^, resp.) (a). CLL cells were CD27^+^ and the majority of them also IgD^+^, with high (b) and low (c) TACI expression.

The clinical and laboratory characteristics of the analyzed patients are presented in Table 2. About half of patients (41, 43.6%) exhibited the most characteristic phenotype of the disease (CD5^+^CD23^+^FMC7^−^CD20^low^sIg^low^CD79b^low^, B-CLL score: 5); among the rest, the majority (43 patients, 45.8%) displayed a B-CLL score of 4, with either FMC7^+^, or sIg^high^, or CD79b^high^, while 10 patients (10.6%) had B-CLL score of 3 (CD5^+^CD23^+^ and CD20^high^ and/or sIg^high^ and/or CD79b^high^, and/or FMC7^+^). Moreover, all CLL cells analyzed were double positive for CD27 and sIgD expression (Figure 1), similar with previous reports in the literature [29, 30]. Seven patients (7.5%) displayed monoclonal M-component in their serum (Table 2) and all were negative for the MyD88-L265P mutation [31].

During the follow-up period (median: 38 months, range: 3–68), 20 patients (21.3%) died due to CLL progression and disease complications (Table 2). Therefore, the median survival of the enrolled patients was 58.6 months (range: 3–158). The older age and the severity of the disease (RAI staging system) were significant risk factors affecting the disease outcome (*P* < 0.001 and *P* = 0.024, resp.). Additional risk factors for a worse outcome were the development of monoclonal gammopathy (*P* = 0.033) (Supplementary Figure 1; see Supplementary Material available online at http://dx.doi.org/10.1155/2015/478753) and the presence of hypogammaglobulinemia (with total immunoglobulin levels below 600 mg/dL, *P* = 0.020) (Supplementary Figure 1), along with the emergence of infections (*P* = 0.020), especially those of the respiratory track (*P* = 0.013).

All CLL patients were also analyzed for IGHV mutational status and CD38 expression; 35 patients (37.6%) displayed unmutated IGHV and 59 (62.8%) were mutated (with SHM greater or equal to 2% different from the germline V gene sequence). Among the latter group (M-CLL), 4 patients displayed borderline IGHV status with SHM between 97 and 98%; therefore, using as cut-off the SHM greater or equal to 3%, U-CLL patients were 39 (42.5%) and M-CLL 55 (57.5%). The pattern of IGHV mutational status of our CLL patients was similar with previous studies [32] and is presented in detail in Supplementary Figure 2.

The most frequent IGHV genes in our cohort of patients were IGHV1-69 (14 patients), IGHV1-2 (7), IGHV3-23 (7), IGHV3-30 (6), and IGHV3-33 (7) (total 41 patients, 43.6%). Within the U-CLL group, 16 CLL cells (41.0%) used a member of the IGHV1 family, with the IGHV1-69 to be the most common type (accounted for 30.8% of all U-CLL patients and 75.0% of U-CLL patients using IGHV1 genes). Within the M-CLL group, only 14 CLL cells (25.5%) used a member of the IGHV1 family, whereas 33 of malignant cells (60.1%) used a member of the IGHV3 family.

Although U-CLL patients (with a SHM cut-off of 2%) exhibited a rather worse outcome compared to M-CLL ones, the difference was not reached to be significant (*P* = 0.191); however, when the SHM cut-off was considered as 3%, the IGHV mutational status was significantly associated with prognosis (*P* = 0.022) (Supplementary Figure 2).

Moreover, the proportion of leukemic cells expressing CD38 above isotype control level ranged from 0.0% to 82.2% with a median of 1.8% and a mean of 11.4%. Subsequently, different cut-offs were considered to define the CD38 positivity; thus, 27 patients (28.7%) displayed CD38 expression above 10%, 21 (22.3%) above 20%, and 12 (12.8%) above 30%. However, in our cohort of patients, CD38 expression was not found to affect the disease outcome (*P* > 0.05, in all cases).

Finally, an overview of PB immunophenotyping of 19 healthy individuals with leukocyte and lymphocyte subpopulations is presented in Supplementary Table 1.

### 3.2. TACI Expression

The immunophenotyping on B-cell subpopulations of healthy individuals indicated that TACI is expressed only on switched and nonswitched memory B cells (CD27^+^IgD^−^ and CD27^+^IgD^+^, resp.) (Figure 1), in accordance with previous reports [33]. On the other hand, the proportion of CLL patients expressing TACI above isotype control level ranged from 0.1% to 57.8% (median: 7.4%, interquartile range [IQR]: 2.1–20.8). Interestingly, the great majority of CLL patients (69, 75.0%) presented with very low TACI expression (<20%) and only 14 patients (14.9%) displayed TACI >30%. Further molecular analyses revealed that the mRNA* TNFRSF13B* expression was equivalent to protein membrane expression (Supplementary Figure 3), indicating that the low TACI expression observed in the majority of CLL cells is due to a dysregulated* TNFRSF13B* gene expression. Considering that all CLL cells were CD27^+^ and the majority of them also IgD^+^ (Figure 1), TACI expression was not correlated with CD27 and IgD.

**Figure 2:**
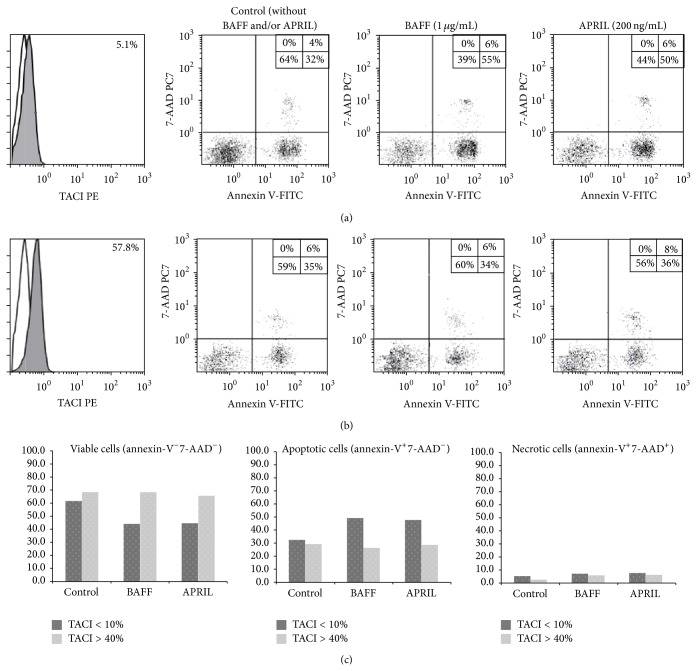
TACI expression protects CLL cells from apoptosis in vitro. CLL cells with very low (a), or high (b) TACI expression were cultured with IMDM medium without any stimulus (internal control) or stimulated by either BAFF or APRIL for 24 h. Cells were harvested and stained with Annexin V (*x*-axis) and 7-AAD (*y*-axis) to discriminate between viable (double negative), apoptotic (Annexin V-FITC positive, 7-AAD negative), and necrotic (double positive) cells. Results are summarized in the bar graphs in the lower panel of the figure (c) and are obtained from three independent experiments in 6 CLL patients with low (3 patients; mean: 3.2%, range: 1.7%–5.1%) and high TACI expression (3 patients; mean: 49.7%, range: 40.1%–57.8%).

To further characterize the effect of TACI on CLL cells, we investigated whether BAFF or APRIL could enhance the survival of CLL cells in vitro. We demonstrated that CLL cells with high TACI expression (>40%) displayed a better survival capacity compared to CLL cells with low to undetectable TACI expression, when cultured in the presence of BAFF and/or APRIL (Figure 2). These results indicate that TACI signaling protects CLL cells from apoptosis in vitro.

In addition, we investigated whether TACI expression on malignant cells is associated with CLL clinical and/or laboratory phenotype. We identified that TACI expression was significantly associated with CD11c expression and the presence of monoclonal M-component. In particular, we observed that TACI expression >20% and a high CD11c (>70%) were mutually exclusive events (*P* = 0.008), while patients with monoclonal gammopathy have higher TACI expression on malignant B cells compared to patients without monoclonal M-component (mean ± SD: 26.2 ± 17.3 versus 12.6 ± 14.7, *P* = 0.020; confirmed also by ROC curve analysis: AUC = 0.765, 95% CI: 0.626–0.964). Further logistic regression analysis, after adjustment for patient gender and age, revealed that TACI expression on CLL cells >10% was significantly associated with a 6.9-fold probability of the presence of monoclonal M-component (OR: 0.866, 95% CI: 1.211–38.925, *P* = 0.030). Finally, no significant associations of TACI expression with autoimmune manifestations (clinical and/or laboratory), the presence of hypogammaglobulinemia (and its severity), the emergence of infections, and the overall survival were found (*P* > 0.05 in all cases).

At the end, we investigated whether TACI expression is correlated with the well-established prognostic factors of CLL, namely, CD38 expression and IGHV mutational status [3, 4, 34], but no significant associations were found (*P* > 0.05 in all cases).

### 3.3. BAFF and APRIL Expression and Their Correlations with Disease Phenotype

**Figure 3:**
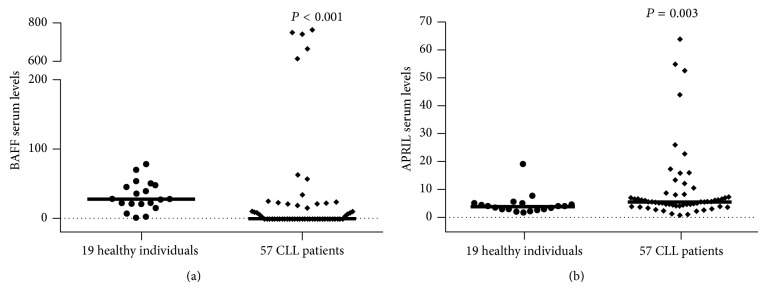
(a) BAFF levels in CLL patients and healthy controls. (b) APRIL serum levels in CLL patients and controls. Lines indicate median values. The significant *P* values refer to Mann-Whitney *U* test.

We demonstrated that CLL patients displayed significantly lower levels of serum BAFF compared to healthy controls (median, IQR: 0 pg/mL, 0–20.7 versus 28.3 pg/mL, 21.6–48.1; *P* ≤ 0.001) (Figure 3), while the majority of patients (30 out of 57 analyzed, 52.6%) exhibited undetectable levels using a hypersensitive BAFF detection kit. Interestingly, 5 patients displayed very high levels of BAFF in their serum (Figure 3) and 3 of them also displayed severe autoimmune manifestations (autoimmune hemolytic anemia, lupus like symptoms with high levels of antinuclear and antiphospholipid antibodies, and vasculitis with high levels of antinuclear and anti-neutrophil cytoplasmic antibodies, resp.). No correlation of BAFF levels with other clinical and laboratory characteristics of CLL patients were found (*P* > 0.05, in all cases).

On the other hand, CLL patients displayed significantly increased levels of serum APRIL compared to healthy controls (median, IQR: 5.1 ng/mL, 3.7–7.8 versus 3.5 ng/mL, 2.5–4.7; *P* = 0.003) (Figure 3). Interestingly, serum APRIL levels were positively correlated with the number of peripheral blood B cells and the percentage of bone marrow infiltration (*r* = 0.305, *P* = 0.023, and *r* = 0.312, *P* = 0.019, resp.) and inversely with IgA and IgG serum levels (*r* = −0.390, *P* = 0.004, and *r* = −0.284, *P* = 0.037, resp.) and the expression of FMC7 (*r* = −0.303, *P* = 0.023).

Finally, no significant correlations of BAFF and APRIL serum levels with TACI expression on CLL cells were observed.

## 4. Discussion

Our study provides clear evidence that CLL cells are characterized by variable TACI expression (with the majority of cases displaying low expression), which was positively correlated with the presence of monoclonal gammopathy and inversely with CD11c expression. However, no prognostic significance of TACI expression in CLL was found, although TACI signaling seems to protect CLL cells from apoptosis in vitro. Finally, we identified that CLL patients displayed low to undetectable BAFF and increased APRIL serum levels compared to healthy individuals, similarly with previous reports [35–38].

To the best of our knowledge this is the largest study analyzing the role of TACI expression in CLL. It is notable that the initial studies reported a rather high TACI expression in the majority of CLL patients; however, the number of the enrolled patients was low, ranged from only 9 to 23 [9, 11, 13]. In a subsequent larger study, Bojarska-Junak et al. analyzed 62 CLL patients and demonstrated that the majority of them displayed a low to undetectable TACI expression [19], similarly with our results. Moreover, they suggested a rather prognostic significance of TACI expression in CLL, since they reported a correlation of TACI with CD38 and ZAP-70 expression [19]. However, such correlations were not confirmed in our study and TACI expression was not associated with the most important marker of prognosis in CLL, namely, the IGHV mutational status. At the end, our study also confirms the findings of Kern et al. that TACI expression protects CLL cells from apoptosis in vitro [11].

Previous studies have reported that gene expression signatures of CLL cells were similar to those observed for memory B cells and marginal zone (MZ) or MZ-like B cells, irrespective of* IGHV* mutational status, suggesting a common mechanism of transformation and/or cell of origin [39, 40]. In this context, we actually demonstrated that CLL cells express CD27^+^, irrespective of their mutational status. However, CLL cells display a variable expression of TACI; this is an unexpected finding since normal CD27^+^ memory B cells are characterized by constitutive TACI expression [33]. After that, the notion that the origin of CLL cell is a memory B cell is rather disputable.

The abovementioned argument (that CLL cell is not a memory B cell) is also supported by the recent results of Seifert et al., suggesting that CLL cells derived from CD5^+^ B-cell populations, already found in young healthy adults, and characterized by deregulated molecules and pathways compared with normal CD5^+^ or conventional B cells. Considering also the major disease subsets, these researchers suggest that U-CLL derives from unmutated mature CD5^+^ B cells, while M-CLL derives from a distinct, previously unrecognized CD5^+^CD27^+^ postgerminal center and antigen-exposed B-cell subset [41]. Along these lines, it can be assumed that CD27 and TACI represent a part of deregulated proteins during transformation, similarly with other known transcription factors and proteins that are necessary for the survival and development of healthy B cells.

Despite the fact that our findings can support the abovementioned model in regard to the origin of CLL cell, the possibility that this cell is derived from a virgin B-cell cannot be excluded. In such a case, the phenotypic and molecular characteristics we detected might result either from activation of mutational machinery (possibly against auto-antigens) or from modifications (transcriptional and/or posttranscriptional) of the expressed molecules. The deregulatory events of emerging subclones could therefore affect their survival capacity (as on TACI expression) and their final predominance.

Over the recent years, it became increasingly clear that external signals from the leukemia microenvironment make pivotal contributions to CLL pathophysiology and progression [42, 43]. This microenvironment includes both BAFF and APRIL, which can rescue highly purified leukemic CLL cells from spontaneous apoptosis in vitro and, thus, may contribute to their prolonged survival in vivo [44]. Therefore, the targeting of BAFF/APRIL receptors could represent an ideal treatment approach. In this context, considering that only a minority of CLL patients displays a high TACI expression and previous studies have shown that BCMA is rather absent on CLL cells [9, 13], the novel anti-APRIL therapeutic approaches should be tested only after the demonstration of TACI expression on CLL cells.

## 5. Conclusions

Our study provides further evidence on the role of TACI expression in CLL. In particular, we demonstrated that there is a remarkable interindividual variability of TACI expression in CLL, although the majority of patients display low to undetectable TACI. Moreover, CLL cells expressing TACI display a better survival capacity in vitro, suggesting that TACI detection might be useful in the selection of patients for novel targeting therapeutic approaches.

## Supplementary Material

Supplementary Figure 1. Cumulative survival of CLL patients of the study according to the presence of (A) autoimmune manifestations (clinical and/or laboratory), and (B) monoclonal M-component.
Supplementary Figure 2. Frequency of specific IGHV genes identified in the patients of the study, according to their mutational status (A) (somatic hypermutation greater or equal to 3% different from the germline V gene sequence is considered as mutated, whereas less than 3% difference is considered as unmutated; details about the cut-off used are presented in the text). Cumulative survival of CLL patients of the study according to their mutational status (B).
Supplementary Figure 3. Comparison of mRNA and protein TNFRSF13B/TACI expression (mean values) in 19 CLL patients of the study.
Supplementary Table 1. Immunophenotyping of 19 healthy individuals analyzed in this study.

## Figures and Tables

**Table 1:** Primers and PCR conditions for the amplification of the analyzed genes.

Gene	Primers	Sequence	PCR conditions

*TNFRSF13B *	Forward Reverse	5′-TCTGCCTGTGTGCCGTCCTC-3′ 5′-CGGCTTCCATCGCGTGAT-3′	95°C for 2 min, followed by 40 cycles (95°C for 10 s, 64°C for 60 s)

*B2M *	Forward Reverse	Commercially obtained by Qiagen, Cat. No PPH01094E	95°C for 10 min, followed by 40 cycles (95°C for 15 s, 60°C for 60 s)

*IGHV* A′ PCR	FR2A FR1C LGH	5′-TGG(A/G)TCCG(C/A)CAG(G/C)C(T/C)(T/C)C(A/G/T/C)-3′ 5′-AGGTGCAGCTG(C/G)(A/T)G(CG)AGTC(A/G/T)GG-3′ 5′-TGAGGAGACGGTGACC-3′	95°C for 2 min, followed by 5 cycles (95°C for 30 s, 63°C for 30 s, 72°C for 30 s), then by 25 cycles (95°C for 30 s, 57°C for 30 s, 72°C for 30 s), and a final elongation at 72°C for 5 min

*IGHV* nested PCR	FR2A VH1 VH2 VH3 VH4 VLJH	Similar with A′ PCR 5′-ACTAGTCGACCTCAGTGAAGGT-3′ 5′-ACTAGTCGACGTCCTGVGCTGGTGAAA(G/C)CCACAC-3′ 5′-ACTAGTGACGGGTCCCTGAGACTCTCCTGTGCAG-3′ 5′-ACTAGTCGACCCTGTCCCTCACCTGC(A/G)CTGTC-3′ 5′-GTGACCAGGGT(A/G/C/T)CCTTGGCCCCAG-3′	95°C for 2 min, followed by 30 cycles (95°C for 30 s, 63°C for 30 s, 72°C for 30 s), and a final elongation at 72°C for 5 min

B2M: beta-2-microglobulin; IGHV: immunoglobulin heavy chain variable region.

**Table 2:** Overview of clinical and laboratory characteristics of the patients of the study.

	Total	At diagnosis	During follow-up	
			No treatment	Relapse^1^

No (%)	94 (100.0)	54 (57.4)	21 (22.3)	19 (20.3)
Gender (M/F)	53/41	34/20	10/11	9/10
Age (y, mean ± SD)	68.6 ± 10.0	68.7 ± 10.5	65.7 ± 9.9	71.3 ± 7.8

At enrollment				
WBC (×10^9^/L) (median, IQR)	22.0 (13.8–34.2)	19.0 (14.2–31.4)	21.0 (19.4–31.7)	34.5 (14.2–61.5)
B cells (×10^9^/L) (median, IQR)	16.0 (5.7–27.1)	9.9 (5.3–20.2)	11.8 (5.2–20.0)	23.6 (5.4–39.9)
Rai staging system				
Stage O (*n*, %)	42, 44.7	28, 51.9	8, 38.0	6, 31.6
Stage I (*n*, %)	22, 23.4	9, 16.6	10, 47.7	3, 15.8
Stage II (*n*, %)	11, 11.7	7, 13.0	2, 9.5	2, 10.5
Stage III (*n*, %)	10, 10.6	8, 14.8	0, 0	2, 10.5
Stage IV (*n*, %)	9, 9.6	2, 3.7	1, 4.8	6, 31.6
Immunophenotyping				
CD5 (median, IQR)	97.5 (86.8–99.4)	95.6 (83.3–98.9)	98.6 (97.1–99.7)	99.3 (82.4–99.7)
CD23 (median, IQR)	91.1 (81.8–96.5)	92.1(84.4–97.0)	93.8 (84.1–97.5)	84.6 (81.5–91.0)
CD20 (median, IQR)	92.0 (83.8–97.5)	93.0 (84.4–98.3)	92.5 (85.3–97.9)	91.0 (75.4–95.9)
CD79b (median, IQR)	92.4 (82.7–98.8)	92.7 (80.8–98.2)	96.4 (84.0–99.9)	88.5 (81.8–98.8)
CD43 (median, IQR)	93.3 (84.6–97.1)	92.8 (81.4–97.0)	94.2 (86.8–97.6)	94.7 (80.6–97.1)
FMC7 (median, IQR)	42.7 (28.9–59.4)	44.5 (30.7–61.2)	44.2 (30.8–61.6)	35.8 (15.0–58.9)
CD38 (median, IQR)	1.8 (0.6–15.5)	1.4 (0.5–8.6)	1.7 (0.7–19.1)	12.0 (0.2–27.1)
CD11c (median, IQR)	34.6 (17.4–52.0)	34.7 (17.4–50.7)	37.1 (20.1–62.1)	28.6 (11.9–55.1)
CD10 (median, IQR)	10.0 (4.8–17.7)	8.7 (4.7–16.4)	11.0 (5.3–19.3)	11.3 (3.8–18.7)
Kappa (*n*, %)	61, 65.0	36, 66.6	15, 71.4	10, 52.6
Lambda (*n*, %)	33, 35.0	18, 33.3	6, 28.6	9, 47.4
Hypogammaglobulinemia				
Total Igs < 600 mg/dL	15, 16.0	4, 7.4	3, 14.3	8, 42.9
Total Igs < 300 mg/dL	3, 3.2	0, 0	0, 0	3, 15.8
Monoclonal M-component^2^	7, 7.4	2, 3.7	2, 9.5	3, 15.8

At enrollment and during follow-up				
Autoimmune/autoinflammatory manifestations	18, 19.1	10, 18.5	1, 4.8	7, 36.8
Coombs direct (*n*, %)^3^	7, 7.4	4, 7.4	0, 0	3, 15.8
Other (*n*, %)^4^	11, 11.7	6, 11.3	1, 4.8	4, 21.0
Infections (*n*, %)^5^	19, 20.2	8, 14.8	1, 4.8	10, 52.6
Respiratory (*n*, %)	10, 10.6	3, 5.5	1, 4.8	6, 31.6
Urinary (*n*, %)	2, 2.1	0, 0	0, 0	2, 10.5
Herpes viruses (*n*, %)^6^	5, 5.3	2, 3.7	0, 0	3, 15.8
Other (*n*, %)^7^	5, 5.3	3, 5.5	0, 0	2, 10.5
Death (*n*, %)	20, 21.3	11, 20.3	2, 9.5	7, 36.8
Survival (mo) (median, IQR)	52.5, 38.5–72.2	43.5, 11.5–53.2	73.0, 52.5–115.5	93.0, 59.0–134.0

Abbreviations: F, female; Igs; immunoglobulins; IQR, interquartile range; M, male; mo, months; SD, standard deviation; WBC, white blood cell count.

^1^Patients at relapse had received in the past chlorambucil-based medication (10 patients), fludarabine-based medication (3), only corticosteroids (1), cyclophosphamide/vincristine-based medication (2), or multiple consecutive treatments (3). Anti-CD20 (rituximab) treatment had been administered in 6 patients, in combination with other medications; ^2^IgG (5 patients), IgM (2 patients).

^3^a patient at diagnosis and another at relapse developed also autoimmune hemolytic anemia.

^4^Including rheumatic polymyalgia (one patient), Crohn disease (one patient), thrombopenic purpura (one patient), hyperthyreodism (antithyroglobulin/anti-TG antibodies; one patient), while 7 patients displayed autoantibodies without clinical signs of autoimmunity (antinuclear/ANA, and/or anti-neutrophil cytoplasmic/ANCA, and/or anti-smooth muscle/ASMA, and/or anti-extractable nuclear/ENA, and/or antiphospholipid/APA antibodies).

^5^3 patients at relapse displayed infections at multiple locations; ^6^herpes simplex (a patient at diagnosis) and varicella zoster virus (4 patients); ^7^including sepsis (2 patients), encephalitis (1), salmonellosis (1) and tuberculosis (1).
