# Supplementary material for: TACI Expression and Signaling in Chronic Lymphocytic Leukemia
Source: J Immunol Res. 2015 Apr 9;2015:478753. doi: 10.1155/2015/478753 (PMC4408744; doi:10.1155/2015/478753)
Supplement: Supplementary file 1 — Supplementary Figure 1. Cumulative survival of CLL patients of the study according to the presence of (A) autoimmune manifestations (clinical and/or laboratory), and (B) monoclonal M-component. Supplementary Figure 2. Frequency of specific IGHV genes identified in the patients of the study, according to their mutational status (A) (somatic hypermutation greater or equal to 3% different from the germline V gene sequence is considered as mutated, whereas less than 3% difference is considered as unmutated; details about the cut-off used are presented in the text). Cumulative survival of CLL patients of the study according to their mutational status (B). Supplementary Figure 3. Comparison of mRNA and protein TNFRSF13B/TACI expression (mean values) in 19 CLL patients of the study. Supplementary Table 1. Immunophenotyping of 19 healthy individuals analyzed in this study. [file 478753.f1.pdf]

A.

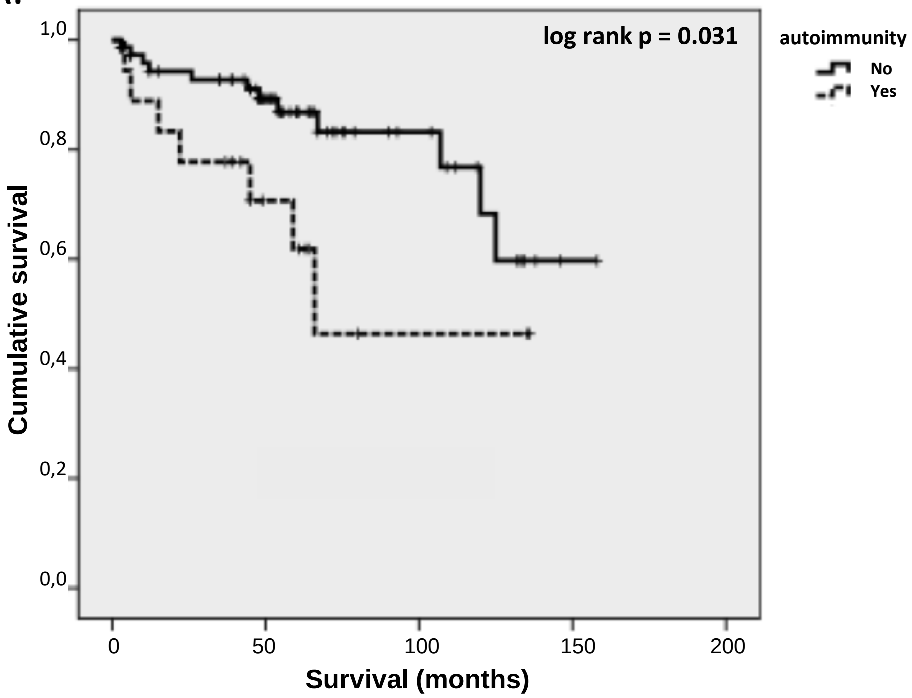
log rank p = 0.031
autoimmunity
No
Yes
1,0
0,8
0,6
0,4
0,2
0,0
Cumulative survival
0
50
100
150
200
Survival (months)

B.

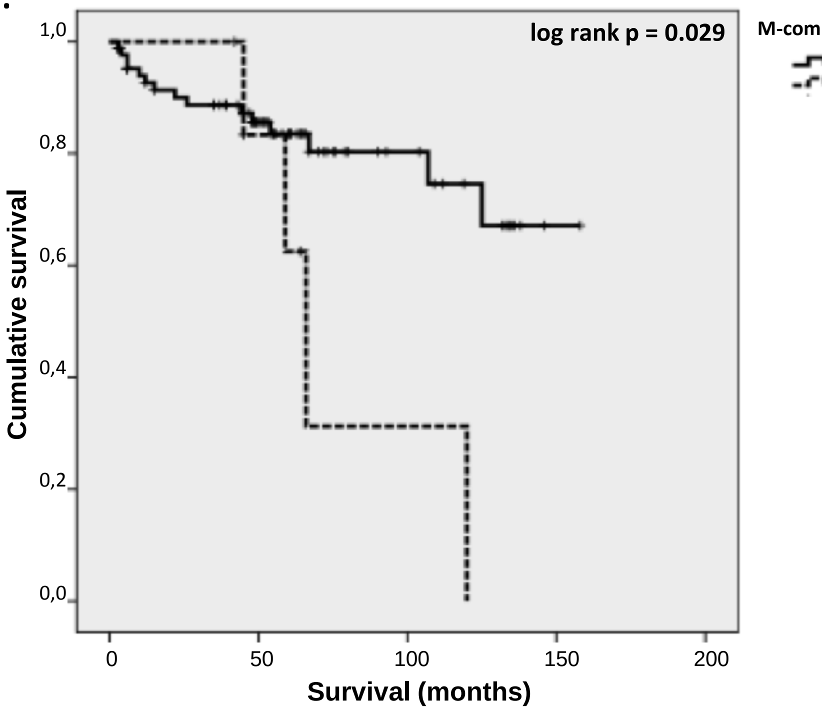
log rank p = 0.029
M-com
1,0
0,8
0,6
0,4
0,2
0,0
Cumulative survival
0
50
100
150
200
Survival (months)

A.

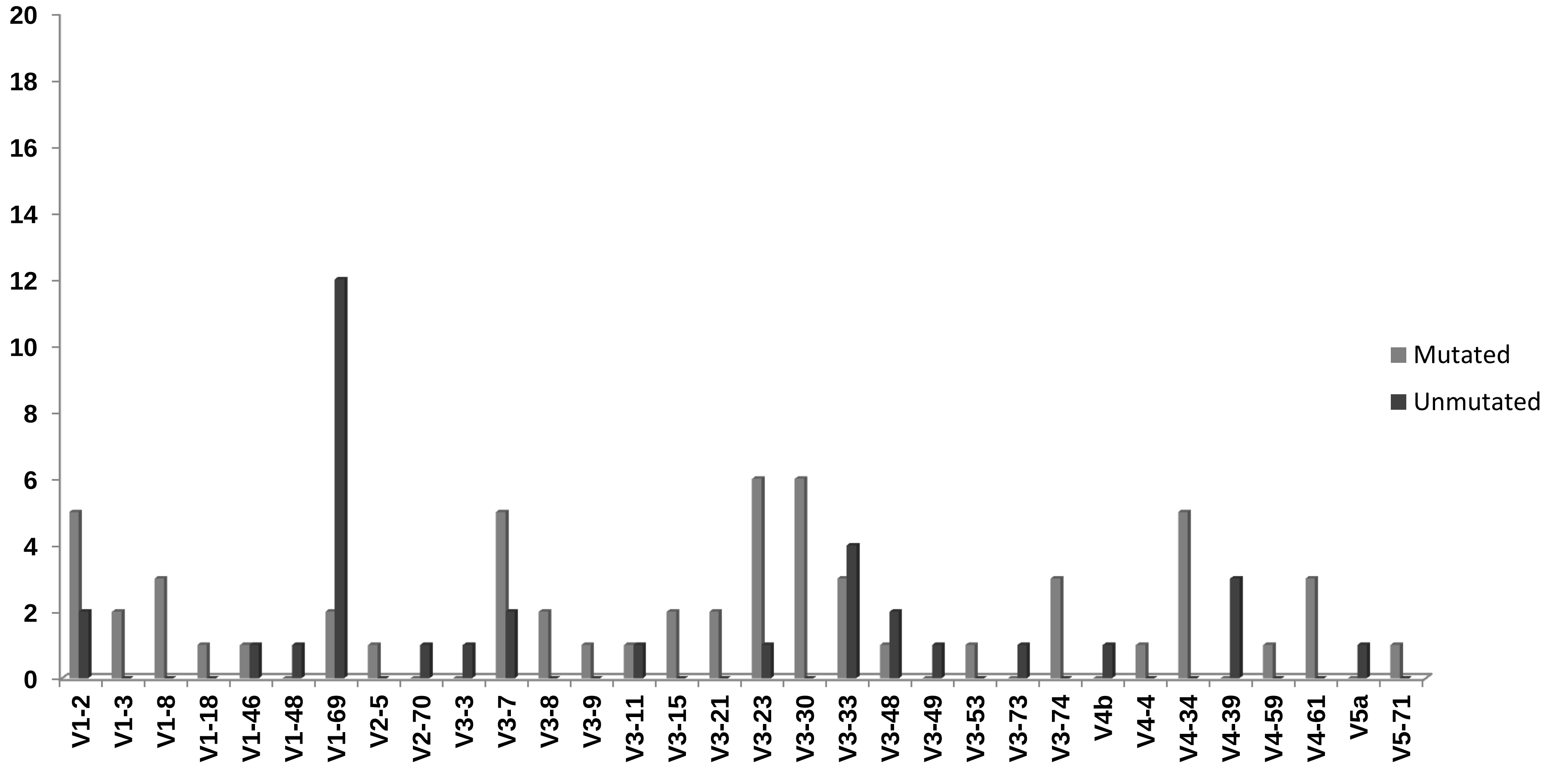

20
18
16
14
12
10
8
6
4
2
0
V1-2
V1-3
V1-8
V1-18
V1-46
V1-48
V1-69
V2-5
V2-70
V3-3
V3-7
V3-8
V3-9
V3-11
V3-15
V3-21
V3-23
V3-30
V3-33
V3-48
V3-49
V3-53
V3-73
V3-74
V4b
V4-4
V4-34
V4-39
V4-59
V4-61
V5a
V5-71
Mutated
Unmutated

B.

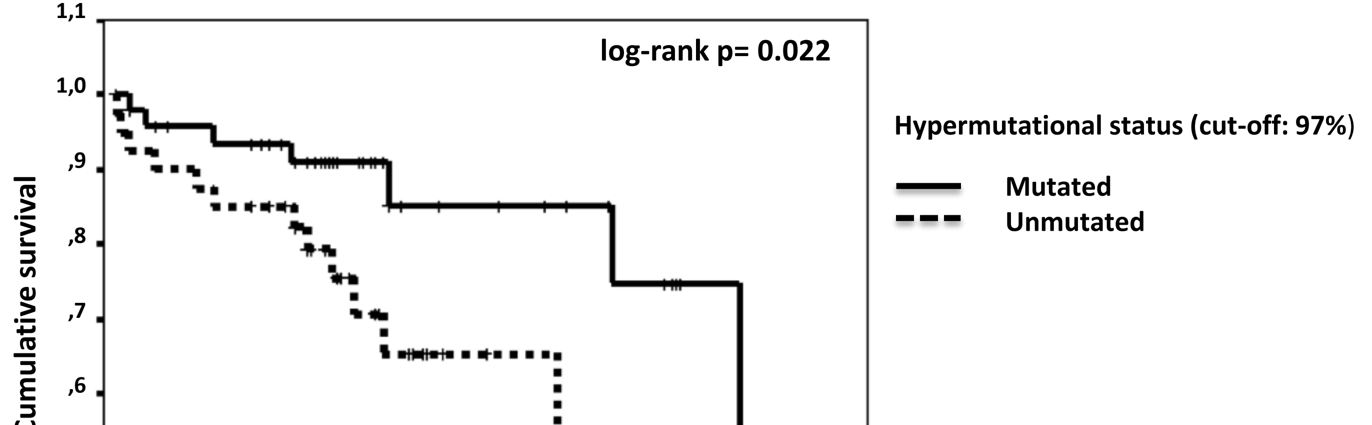

1,1
1,0
,9
,8
,7
,6
Cumulative survival
log-rank p= 0.022
Hypermutational status (cut-off: 97%)
Mutated
Unmutated

Supplementary Table 1. Immunophenotyping of 19 healthy individuals analyzed in this study

WBC (x 10^9/L) (median, IQR)	6.60 (5.38, 8.23)
Neutrophils (x 10^9/L) (median, IQR)	3.70 (2.87, 4.99)
Monocytes (x 10^9/L) (median, IQR)	0.39 (0.28, 0.49)
Lymphocytes (x 10^9/L) (median, IQR)	2.34 (1.88, 2.92)
T cells (%) (median - IQR)	68.30 (61.22, 75.97)
T cells (x 10^9/L) (median - IQR)	1.53 (1.25, 1.96)
T4 cells (%) (median - IQR)	65.85 (58.45, 71.45)
T4 cells (x 10^9/L) (median - IQR)	1.00 (0.81, 1.26)
T8 cells (%) (median - IQR)	31.30 (25.67, 37.47)
T8 cells (x 10^9/L) (median - IQR)	0.48 (0.35, 0.64)
Ratio T4/T8 (median, IQR)	1.20 (0.91, 2.00)
B cells (%) (median, IQR)	9.60 (7.30, 12.37)
B cells (x 10^9/L) (median, IQR)	0.22 (0.16, 0.32)
$IgD^+/CD27^-$ (%) (median, IQR)	61.85 (50.90-76.45)
$IgD^+/CD27^-$ (x 10^9/L) (median, IQR)	0.12 (0.09-0.19)
$IgD^+/CD27^+$ (%) (median, IQR)	9.10 (4.82-14.12)
$IgD^+/CD27^+$ (x 10^9/L) (median, IQR)	0.02 (0.01-0.04)
$IgD^-/CD27^+$ (%) (median, IQR)	20.20 (12.60-30.48)
$IgD^-/CD27^+$ (x 10^9/L) (median, IQR)	0.04 (0.02-0.07)

Abbreviations: IQR, interquartile range; WBC, white blood cell count

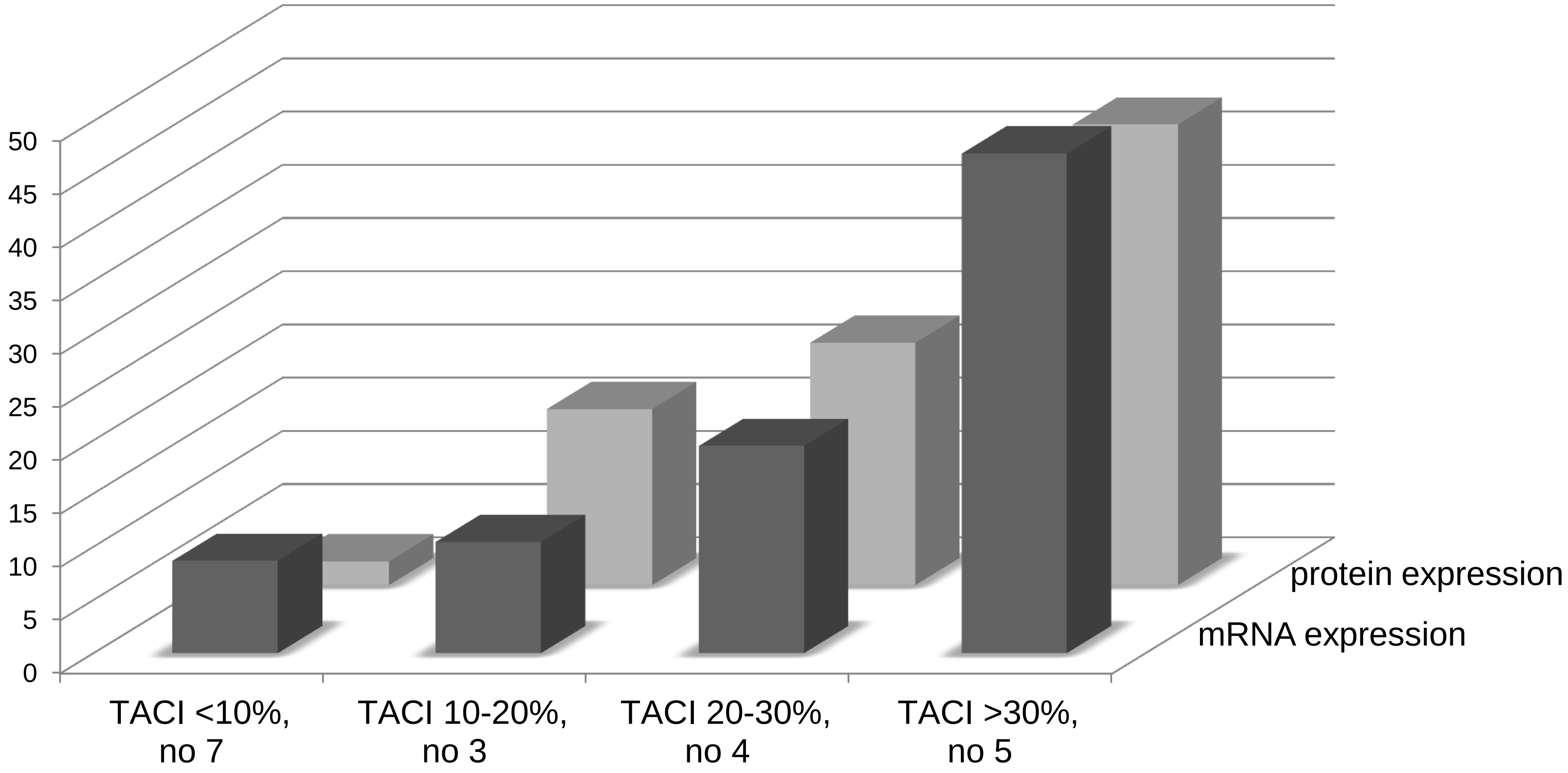

0
5
10
15
20
25
30
35
40
45
50
protein expression
mRNA expression
TACI <10%, no 7
TACI 10-20%, no 3
TACI 20-30%, no 4
TACI >30%, no 5
